# Significance of myocardial flow reserve after revascularization

**DOI:** 10.1093/ehjci/jead151

**Published:** 2023-07-08

**Authors:** Antti Saraste, Teemu Maaniitty

**Affiliations:** Heart Center, Turku University Hospital and University of Turku, Hämeentie 11, 20520 Turku, Finland; Turku PET Centre, Turku University Hospital and University of Turku, Turku, Finland; Turku PET Centre, Turku University Hospital and University of Turku, Turku, Finland; Department of Clinical Physiology, Nuclear Medicine and PET, Turku University Hospital, Turku, Finland

**This editorial refers to ‘Prognostic value of global myocardial flow reserve in patients with history of coronary artery bypass grafting’, by M. Al Rifai *et al.*https://doi.org/10.1093/ehjci/jead120.**

Patients remain at risk of cardiovascular events after coronary artery bypass graft surgery (CABG) that is partly related to the progression of coronary artery disease (CAD) in native coronary arteries and bypass grafts.^1^ In patients with recurrent symptoms after revascularization, stress imaging can reveal myocardial ischaemia as the underlying cause of symptoms and provides prognostic information. In a meta-analysis including a total of 12 874 symptomatic or asymptomatic patients with a history of revascularization, abnormal results at nuclear myocardial perfusion imaging (MPI) or stress echocardiography were associated with an average of two-fold risk of cardiac events.^2^ More recently, the presence of inducible ischaemia on stress perfusion cardiac magnetic resonance was found an independent predictor of cardiovascular events after CABG.^3^

Positron emission tomography (PET) myocardial perfusion imaging accurately detects obstructive CAD. Measurement of myocardial blood flow (MBF) in absolute units (mL/min/g of myocardial tissue) using PET can improve the diagnostic evaluation of CAD over relative perfusion differences.^4^ Furthermore, impaired MBF and myocardial flow reserve (MFR) in response to vasodilator stress is a strong prognostic marker.^5^ In patients with prior CABG, the ischaemic burden on PET provides prognostic information in predicting all-cause mortality and cardiac deaths.^6^ However, the prognostic value of MFR assessed by PET is unclear in this population.

Al Rifai *et al*.^7^ report the prognostic value of MFR in a cohort of 836 patients with a history of CABG who underwent rest-stress ^82^Rb PET MPI due to a clinical indication for evaluation of ischaemia. Up to 66% of patients had MFR <2, a pre-defined cut-off value for abnormal MFR.^4^ Patients with abnormal MFR were older and more often men, hypertensive and diabetic, had lower left ventricular ejection fraction (LVEF), and were more likely to have scar or regional inducible ischaemia. Over a median follow-up time of 12 months, 122 patients suffered an adverse event, including 46 heart failure admissions, 28 all-cause deaths, 23 myocardial infarctions, and 25 unplanned late revascularizations. Reduced MFR was a predictor of outcome in adjusted analyses on top of early revascularization, clinical variables, and other PET parameters, including LVEF, LVEF reserve, regional perfusion defects, and transient ischaemic dilatation. Patients with abnormal MFR had a higher rate of the composite outcome (hazard ratio 2.06, 95% confidence interval 1.23–3.44) than those with normal MFR; the cumulative incidence of events being 18.4 vs. 7.1%.

Impaired MFR may be due to obstructive epicardial CAD, diffuse CAD, or microvascular dysfunction.^8^ In the study of Al Rifai *et al*., both regional ischaemia and abnormal MFR were common findings in patients with a history of CABG.^7^ However, there was no interaction between ischaemia and MFR in predicting the outcome, and MFR was prognostic irrespective of the presence of scar. Thus, regional perfusion abnormalities and MFR may identify different prognostic phenotypes and have implications for the selection of therapy. Observational studies have found that the reduced MFR may identify patients with survival benefit from early revascularization when compared with medical therapy.^9^ Such a study requires a large patient cohort and remains to be studied in patients with a history of CABG in whom the decision to proceed with repeat revascularization requires careful weighing of the risks and potential benefits. The use of evidence-based medical therapy is strongly associated with favourable outcomes after myocardial revascularization,^10^ and there is also an interest in reduced MFR as a potential target for novel therapies.^8^

In patients with a history of CABG, incomplete revascularization, occlusion of bypass grafts, and progression of CAD in the native coronary arteries can contribute to the presence of residual ischaemia. Coronary computed tomography angiography (CTA) has been shown to accurately identify stenosis in both venous and arterial bypass grafts. The presence of coronary artery territories supplied by an obstructed native coronary artery in the absence of patent bypass graft, or obstruction of a coronary artery distal to the graft insertion, predicts adverse cardiac events in patients with a history of CABG.^11^ Hybrid imaging with coronary CTA and stress MPI has been shown to provide useful complementary information in patients with a history of CABG enabling the integration of myocardial perfusion abnormalities with the anatomy of subtending coronary arteries or bypass grafts.^12,13^

Coronary microvascular dysfunction often co-exists with epicardial CAD, but is also common in the absence of CAD in patients with various forms of cardiomyopathy and heart failure with either reduced or preserved LVEF.^14^ A study found that impaired MFR is independently associated with diastolic dysfunction and risk of heart failure hospitalization in patients with preserved LVEF.^15^ Notably, in the study of Al Rifai *et al*., reduced MFR showed a particularly strong association with heart failure admissions (hazard ratio 2.92).^7^ Furthermore, both LVEF and MFR were significant predictors of the primary outcome in the adjusted model.

The study of Al Rifai *et al*. extends the evidence regarding the prognostic value of reduced MFR in CAD to patients with a history of CABG, suggesting that MFR may provide incremental risk prediction beyond regional myocardial ischaemia and LVEF. The study encourages further evaluation of the value of MFR in optimizing therapies to improve outcomes after myocardial revascularization.

## Data Availability

No new data were generated or analysed in support of this research.
